# Palmitoylation of synaptic proteins: roles in functional regulation and pathogenesis of neurodegenerative diseases

**DOI:** 10.1186/s11658-024-00625-2

**Published:** 2024-08-10

**Authors:** Jiaying Peng, Danchan Liang, Zhonghao Zhang

**Affiliations:** 1 Shenzhen Key Laboratory of Marine Bioresources and Ecology, Brain Disease and Big Data Research Institute, College of Life Sciences and Oceanography, Shenzhen University, Shenzhen, China; 2 Shenzhen-Hong Kong Institute of Brain Science-Shenzhen Fundamental Research Institutions, Shenzhen, China

**Keywords:** Palmitoylation, Synaptic proteins, Palmitoyl acyltransferases, DHHC, Neurodegenerative diseases

## Abstract

Palmitoylation is a type of lipid modification that plays an important role in various aspects of neuronal function. Over the past few decades, several studies have shown that the palmitoylation of synaptic proteins is involved in neurotransmission and synaptic functions. Palmitoyl acyltransferases (PATs), which belong to the DHHC family, are major players in the regulation of palmitoylation. Dysregulated palmitoylation of synaptic proteins and mutated/dysregulated DHHC proteins are associated with several neurodegenerative diseases, such as Alzheimer’s disease (AD), Huntington’s disease (HD), and Parkinson’s disease (PD). In this review, we summarize the recent discoveries on the subcellular distribution of DHHC proteins and analyze their expression patterns in different brain cells. In particular, this review discusses how palmitoylation of synaptic proteins regulates synaptic vesicle exocytotic fusion and the localization, clustering, and transport of several postsynaptic receptors, as well as the role of palmitoylation of other proteins in regulating synaptic proteins. Additionally, some of the specific known associations of these factors with neurodegenerative disorders are explored, with a few suggestions for the development of therapeutic strategies. Finally, this review provides possible directions for future research to reveal detailed and specific mechanisms underlying the roles of synaptic protein palmitoylation.

**Supplementary Information:**

The online version contains supplementary material available at 10.1186/s11658-024-00625-2.

## Introduction

The point of contact between two adjacent neurons is known as the synapse, which is responsible for the transfer and transformation of information from one neuron (presynaptic) to the next neuron (postsynaptic). This process is termed as synaptic transmission or neurotransmission [107]. Neurotransmission to postsynaptic receptors is mediated by chemical neurotransmitters packaged in synaptic vesicles (SVs). During this process, many synaptic proteins, including presynaptic and postsynaptic proteins, form specialized structures and perform crucial functions. Presynaptic proteins, particularly soluble *N*-ethylmaleimide-sensitive factor attachment receptor proteins (SNAREs), presented on the plasma membrane and SV membranes, containing vesicle-associated membrane protein (VAMP2, also known as synaptobrevin-2, SYB2), SNAP25, and syntaxin 1 (STX1), are essential for mediating membrane fusion during repeated cycles of SV exocytosis and endocytosis [22, 23, 42, 57, 69, 102, 109, 128]. Upon fusion, neurotransmitters are released by SV exocytosis into the synaptic cleft [56], where they bind to receptors, mainly located in postsynaptic densities (PSDs), i.e., complex molecular machines embedded in the postsynaptic membrane containing a lattice-like array of interacting proteins that organize and stabilize synaptic receptors, ion channels, structural proteins, and signaling molecules required for optimal synaptic transmission and function [11]. Scaffold proteins on the PSD regulate the dynamics of cytoskeletal structures and play major roles in diverse synaptic functions, such as trafficking, anchoring, and clustering of postsynaptic receptors and adhesion molecules [65, 116]. Overall, these findings clearly indicate that these synaptic proteins are highly dynamic and are involved in neurotransmission.

Posttranslational modifications (PTMs) are critical events that occur during and after protein synthesis [1]. PTMs are ubiquitous in synaptic proteins and play significant roles in enabling protein function and determining protein localization. Several excellent studies demonstrated that PTMs could support synaptic protein function and synaptic plasticity, ultimately contributing to better brain functions [1, 19, 39, 59, 76, 118]. PTMs contribute to the normal function of cells, and aberrant protein modification can drive the onset or promote the development and progression of neurodegenerative disorders [24]. Among the different types of PTMs, covalent additions are critical and contribute to the dynamic regulation and functional performance of proteins. Palmitoylation is a lipid modification in which palmitate (a lipid group) is added to cysteine residues via a labile thioester linkage [9, 24]. This type of PTM is widely observed in the regulated trafficking and functional modulation of diverse membrane proteins and membrane-associated signaling factors. Previous studies have documented that palmitoylation of synaptic proteins is critical for maintaining synaptic functions and is closely associated with brain physiology and pathophysiology [7, 32, 93]. For instance, components of SNAREs, including VAMP2, SNAP25, and STX1, as well as Ca^2+^ sensor proteins, synaptotagmins (SYTs), are modified by palmitoylation in neurons, and palmitoylation might contribute to the regulation of synaptic vesicle fusion [85]. Accumulating evidence indicates the crucial roles of palmitoylation-dependent regulation of postsynaptic α-amino-3-hydroxy-5-methyl-4-isox-azoleproprionic acid receptors (AMPARs) in the physiology of synaptic function, plasticity, and learning [43, 101]. Several studies have provided comprehensive information regarding the pivotal roles of synaptic protein palmitoylation in various aspects of pathophysiology. A recent review [12] provided substantial evidence for the association of aberrant palmitoylation with neurological and psychiatric diseases such as Alzheimer’s disease [2, 67, 71, 72, 96], Huntington’s disease [68, 100, 110, 124], intellectual disabilities [70, 88], schizophrenia [75, 84, 122], and major depressive disorders [64, 81]. Therefore, a better understanding of the palmitoylation of synaptic proteins may be helpful in elucidating the mechanisms of neurodegenerative disorders.

Here, we discuss recent discoveries of palmitoylating enzymes (the DHHC family), including their subcellular distribution and expression patterns in neurons, as well as the role of palmitoylation of synaptic proteins in various aspects of pathophysiology, and their association with neurodegenerative disorders.

## Subcellular localization and expression patterns of DHHC family members in brain cells

Palmitoylation is catalyzed by a family of palmitoyl acyltransferases, which are integral membrane enzymes known as DHHC protein acyltransferases (DHHC-PATs) [53, 91, 106]. The term DHHC-PATs has been coined because this family contains the highly conserved Asp–His–His–Cys (D–H–H–C) tetrapeptide motif that is necessary for catalysis. With the discovery of 23 DHHC-PATs, their importance in cellular physiology and disease has become increasingly evident. Each DHHC protein may act on a specific substrate(s), which explains why many DHHC proteins exist in a single organism. In addition, DHHC proteins exhibit distinct intracellular localization and tissue (cell)-specific expression patterns that modulate different functions.

**Fig. 1:**
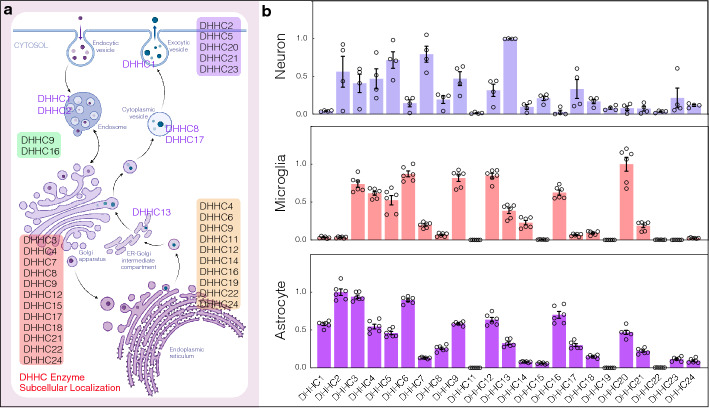
Distribution and expression patterns of the DHHC family in brain cells. **a** Subcellular localization of DHHC proteins, mainly in endoplasmic reticulum (ER) and/or Golgi, and a few localized in the cytoplasm, endosome, mitochondrion, plasma membrane, cytoplasmic vesicle membrane, and SV membrane. **b** Differential expression (RNA) of DHHC proteins in three different primary cell types in the mouse brain (i.e., neurons, microglia, and astrocytes). We performed quantitative real-time PCR to detect DHHC protein expression levels. After normalizing to the reference gene (GAPDH), we determined the relative gene expression levels (*n* = 6 or 4). Table S1 summarizes the primer sequences

Most human DHHC proteins are localized in the endoplasmic reticulum (ER) and/or Golgi, with a few localized in the cytoplasm, endosomes, mitochondria, plasma membranes, cytoplasmic vesicle membranes, and SV membranes [12]. Based on previous research data [31, 79, 89], DHHC2, 4, 9, 12, 13, 22, and 24 are localized both in the ER and Golgi. DHHC6 and 14 are only found in the ER, while DHHC3, 7, 15, and 18 have been reported as Golgi-resident proteins. Several DHHC proteins, including DHHC2, 5, 11, 19, 20, and 21, are localized to the plasma membrane. In addition, DHHC9 and 16 are found in the cytoplasm, whereas DHHC8 and 17 have been identified in the cytoplasmic vesicle membrane. A small number of DHHC proteins, such as DHHC1 and 2, are localized in endosomes. A detailed map of the intracellular distribution of DHHC proteins is shown in Fig. 1 (left). However, most of the current findings are based on co-expression studies. Therefore, more reliable studies on endogenous DHHC proteins are needed. In addition, it is worth noting that DHHC proteins have different intracellular distributions in different cell types. Therefore, it is important to provide comprehensive information regarding their intracellular distribution.

Protein palmitoylation is closely involved in the regulation of physiological functions in the brain. Many studies have reported that DHHC proteins exhibit cell-specific expression patterns in different brain regions to modulate various functions [46, 77, 117, 126]. Wild et al. used existing single-cell RNAseq data to develop an interactive network tool for visualizing palmitoylation-regulating gene expression patterns across various cell types [119]. Their data indicate that most DHHC expression levels are low in neurons, microglia, and astrocytes. Here, we measured DHHC protein levels in these primary mouse brain cell types to analyze their differential expression among different brain cells (Fig. 1, right). We found that DHHC2, 3, 4, 5, 7, 9, and 13 proteins were relatively highly expressed compared with other DHHC protein in neurons. In microglia, DHHC3, 4, 5, 6, 9, 12, 16, and 20 proteins were expressed at high levels. In astrocytes, the expression levels of DHHC1, 2, 3, 4, 5, 6, 9, 12, 16, and 20 were relatively higher than those of other proteins. These findings, taken together with the intracellular distribution of these proteins, may provide clues for discovering the specific biological functions of each DHHC protein in brain.

## Synaptic protein palmitoylation and impact on synaptic transmission and functions

Palmitoylation is a reversible PTM; thus, the levels of palmitoylated proteins is regulated through a cycle of palmitoylation and depalmitoylation [121]. Palmitoylation affects the protein’s affinity for membranes and allows proteins to shuttle between intracellular compartments. The nature of protein palmitoylation has a significant influence on intracellular localization, stability, function, and secretion as well as on interactions with other proteins [83]. To date, extensive studies have focused on the palmitoylation of synaptic proteins, and over 40% of all known synaptic proteins have been identified as substrates for palmitoylation [95]. Given the functional diversity of synaptic proteins, it is not surprising that palmitoylation of synaptic proteins has been described as affecting synaptic transmission and functions. Understanding how palmitoylation regulates the function of synaptic proteins and synapses is an important driver of current research in this field. In this section, we discuss palmitoylation of synaptic proteins in various aspects of synaptic pathophysiology.

### Palmitoylation of presynaptic proteins regulates synaptic vesicle exocytotic fusion

**Fig. 2:**
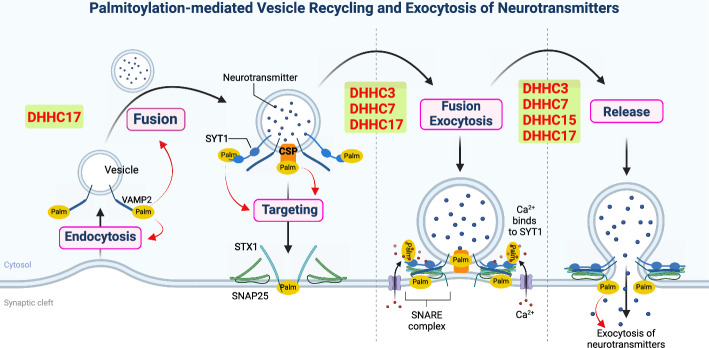
Palmitoylation-mediated vesicle recycle and exocytosis of neurotransmitters. Palmitoylation of presynaptic proteins plays critical roles in regulating synaptic vesicle exocytotic fusion, thus mediating exocytosis and endocytosis of SVs. The palmitoylation of SYT1, CSP, and STX1 mediated by DHHC3, 7, or 17 are important in facilitating SV targeting and membrane attachment. Then the SV exocytotic fusion process is regulated by the palmitoylation of VAMP2 and SNAP25, which are mediated by DHHC3, 7, 15, or 17. In addition to exocytosis, the DHHC17-mediated palmitoylation of VAMP2 is also vital for the fast synaptic-vesicle endocytosis to replenish the pool of SVs for the next round of exocytosis

Neurotransmitter release is restricted to a specialized presynaptic structure called the active zone (AZ), an important sensor of the action potential responsible for docking and priming SVs, as well as triggering the exocytotic fusion of neurotransmitter-filled SVs with the presynaptic plasma membrane [5, 108]. The fusion of SVs with the pre-synaptic plasma membrane is an essential event involving an array of proteins, among which the members of the SNARE complex are of central importance [82]. SNARE proteins interact to form a tight complex that represents the minimal membrane fusion machinery by bridging the SVs and the plasma membrane. Recently, major functional SNARE proteins, including STX1, VAMP2, and SNAP25, were reported to be modified by palmitoylation in neurons [85] (Fig. 2).

#### STX1 and VAMP2

STX1 and VAMP2, two integral transmembrane proteins located on the plasma and SV membranes, respectively, have been demonstrated to be major functional SNARE proteins [85]. The C-terminal transmembrane domain (TMD) and SNARE motif in these proteins are separated only by a short polybasic juxtamembrane domain (JMD). A recent study by Vardar et al. [114] showed that the palmitoylation of the TMD of STX1A plays an important role in modulating vesicle fusion in mammalian neurons. They found that the loss of STX1A palmitoylation inhibited spontaneous vesicle fusion. The JMD of STX1A modifies the palmitoylation of its TMD, which in turn regulates spontaneous neurotransmitter release [114]. However, the DHHC proteins responsible for mediating palmitoylation of STX1A have not been identified. VAMP2 is an SV-associated SNARE protein that regulates intracellular vesicle fusion process [123]. In addition to regulating exocytosis, it is vital for fast synaptic-vesicle endocytosis to replenish the pool of SVs for the next round of exocytosis [17]. DHHC17 is an interesting candidate protein for mediating the palmitoylation of VAMP2. A previous study revealed that recombinant SNAP25 added to SVs competes with VAMP2 for palmitoylation as DHHC17 also modifies SNAP25 [115]. However, the exact role of VAMP2 palmitoylation in trafficking and other functions has not yet been investigated.

#### SNAP25

Palmitoylation of the SNAP25 family has been suggested to mediate its stable association with the membrane and determine the precise intracellular distribution of SNAP25 [32, 35]. This modification of SNAP25 is considered fundamental to its function as it mediates the accumulation of SNAP25 at exocytotic sites. Increased palmitoylation of SNAP25 inhibits its function by reducing regulated exocytosis [92]. SNAP25a and SNAP25b are two major isoforms of SNAP25 that are highly expressed in the brain; SNAP25b promotes exocytosis to a greater extent than done by SNAP25a [104]. SNAP25b is reportedly palmitoylated by DHHC3, DHHC7, and DHHC17 [34]; however, little information is available on the activity of the 23 DHHC protein on SNAP25a and SNAP23.

#### SYTs

It is well known that SV fusion is stimulated by an increase in intraterminal Ca^2+^ levels with the help of major Ca^2+^ sensors, SYTs. A previous study reported that SYTs are efficiently palmitoylated from mature synaptic vesicles [115]. SYT1 is the most well-characterized member of the SYTs family and has been proposed to act as a Ca^2+^ sensor for regulated exocytosis. Kang et al. [52] demonstrated that the reduced presynaptic localization of a palmitoylation-deficient mutant of SYT1 was linked to an increase in the surface expression of SYT1 and reduced endocytosis, implying that palmitoylation of N-terminal cysteines at SYT1 is critical for regulating surface expression and for sorting SYT1 to an intracellular vesicular compartment at presynaptic sites. In addition, the cysteine residues in SYT1 play an important role in oligomerization [114]. Thus, palmitoylation of SYT1 may affect protein interactions and influence its internalization, intracellular sorting, and/or function. Although DHHC proteins that modify SYT1 have not been fully assessed, DHHC17 has been reported to mediate the palmitoylation of SYT1, similar to its action on VAMP2 [49]. As the palmitoylation of VAMP2 and SYT1 is important for regulating their co-clustering on the vesicle membrane, such spatial coordination may optimize the efficiency of fusion [8, 29, 52].

#### Cysteine string protein (CSP)

CSP is an interesting protein that is localized in neuronal synaptic vesicles. It contains a string of ~12–15 cysteine residues, a majority of these which are palmitoylated [36]. Therefore, CSP is likely one of the most palmitoylated proteins in neuronal cells. It has been identified that the Golgi proteins, DHHC3, DHHC7, DHHC15, and DHHC17, are active enzymes for CSP [33]. CSP palmitoylation is essential for its function as it facilitates membrane attachment and SV targeting due to its soluble character [80]. However, as it is a heavily palmitoylated protein, this modification may have additional functions. However, the exact functions of CSP in exocytosis is unknown, and it is difficult to speculate on the precise roles of CSP palmitoylation in the regulation of exocytosis. Accumulating evidence suggests that CSP plays an important role in maintaining the conformation of SNAP25, thereby facilitating the membrane-fusing SNARE complex assembly [6]. Further investigations are required to determine whether CSP palmitoylation regulates exocytosis via protein interactions with SNAP25.

### Palmitoylation of postsynaptic proteins regulate their localization, transport, and clustering

Several types of receptors, such as excitatory glutamate receptors and inhibitory glycine receptors, are localized on the postsynaptic membrane to mediate synaptic transmission. These receptors are folded and assembled within the ER, trafficked to the Golgi, and eventually inserted into the plasma membrane to perform their functions. As previously documented, postsynaptic proteins are more likely to be palmitoylated than most other proteins. In recent years, it has become clear that palmitoylation of these receptors is crucial for their localization, trafficking, and clustering for cell surface stability and function, which is emerging as a key mechanism for synaptic transmission [87, 101, 113].

#### AMPARs and NMDARs

**Fig. 3:**
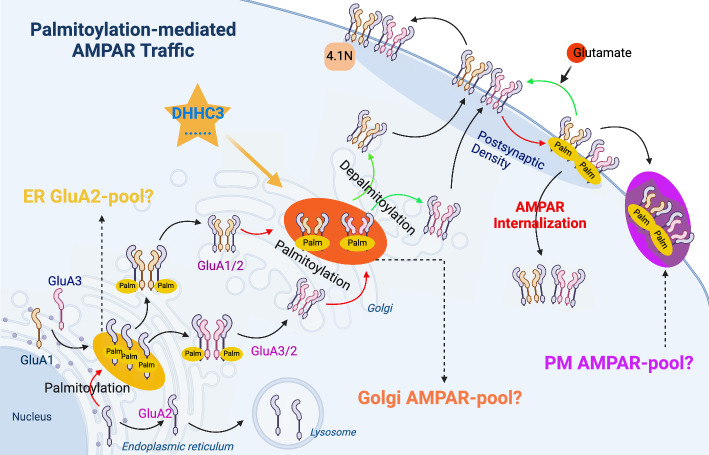
Palmitoylation in regulating AMPAR trafficking. As the AMPAR heteromers are assembled in ER, it is the start site for synaptic trafficking. Palmitoylation of AMPAR2 (GluA2) in the ER is crucial for its sorting to the lysosome for degradation. After trafficking to Golgi, the AMPAR1/2 (GluA1/2) are palmitoylated by DHHC3 to prevent their trafficking to plasma membrane and keep them in Golgi. Interestingly, the depalmitoylation of AMPARs in Golgi is a releasing signal for trafficking of AMPARs from Golgi to the membrane, where the AMPARs are palmitoylated again for the AMPARs internalization. Some functional protein pools may exist in the ER, Golgi, and membrane regions, for triggering the palmitoylation/depalmitoylation process of AMPARs; however, detailed information is lacking

The postsynaptic AMPARs mediate the majority of the fast component of excitatory postsynaptic currents (EPSCs) and are found to be critical for synaptic transmission and plasticity. Many studies have shown that palmitoylation is a key modification of AMPARs that regulates their synaptic expression and localization. AMPARs are composed of four subunits (GluA1–4), each being processed by a distinct machinery that independently determines the trafficking routes of AMPARs and contributes to their synaptic function. AMPAR heteromers are assembled in the ER, which is the start site for synaptic trafficking [37]. Each subunit can be palmitoylated at two conserved Cys residues located next to TMD2 and TMD4 (near the C-terminal domain, CTD). Palmitoylation of GluA2 occurs primarily within the ER, and reduced palmitoylation of GluA2 in the ER prevents its sorting to lysosomes for degradation [125]. However, the PATs localized to the ER during GluA2 palmitoylation remains unclear. After trafficking from the ER to the Golgi, palmitoylation of GluA1 and GluA2, which is mediated by DHHC3 [also known as Golgi-specific DHHC zinc finger protein (GODZ)], results in the accumulation of AMPARs in the Golgi and a reduction in surface expression to ensure correct receptor maturation [43]. It is plausible that depalmitoylation of AMPARs in the Golgi serves as a release signal for the trafficking of AMPARs from the Golgi to the plasma membrane when stimuli are applied. Therefore, based on available data, considering that protein pools containing AMPARs exist in the ER, Golgi, and membrane regions is a reasonable hypothesis (Fig. 3). The formation of these protein pools may be associated with palmitoylation of the corresponding subunits. However, the underlying mechanisms remain unclear and it is still uncertain how the existence of these protein pools affects synaptic plasticity. In addition, palmitoylation of GluA1 at TMD4 inhibits GluA1 interaction with the 4.1N protein and triggers the endocytosis of AMPARs, which appears to act as a “quantitative regulation” of synaptic receptor number by regulating AMPA- and NMDA-induced AMPARs internalization [43].

In contrast with AMPARs, N-methyl-d-aspartic acid receptors (NMDARs) are considered to be more stable in neurons. NMDARs are heteromeric combinations of the GluN1, GluN2, and GluN3 subunits. GluN2A and GluN2B are the major GluN2 subunit types, both of which can be palmitoylated with two distinct clusters of palmitoylation sites (clusters I and II) in their CTD [44]. The CTD of many membrane protein subunits contain ER retention motifs that maintain unassembled and improper oligomers in the ER. However, it has not been reported whether the palmitoylation of GluN2 on clusters I and II could modulate the exit of NMDARs from the ER. However, a previous study revealed a novel regulatory mechanism of NMDARs trafficking involving the palmitoylation of clusters I and II [66]. Palmitoylation of Cys cluster I ensures proper surface delivery and steady-state surface expression, whereas the depalmitoylated form of surface NMDARs is less stably associated with the plasma membrane, thereby controlling the constitutive internalization of NMDARs. Palmitoylation of Cys cluster II leads to accumulation of the receptor in the Golgi, suggesting that Cys cluster II depalmitoylation may trigger receptor release from the Golgi and trafficking to the cell surface, similar to AMPARs. Moreover, Golgi-specific DHHC3 functions as a PAT for palmitoylation of Cys cluster II [44]. These data indicate that the palmitoylation of NR2 subunits is emerging as a regulator of NMDARs trafficking.

#### GABAARs and PSD95

The GABAA receptors (GABAARs) are heteropentameric and are composed mostly of alpha, beta, and gamma1–3 subunits. Among these subunits, gamma2 is of special interest because of the rich cysteine residues in its large intracellular loop, making it a potential candidate for palmitoylation [129]. Several studies have demonstrated that gamma2 can be palmitoylated, which is required for controlling both GABAA receptor clustering at synaptic sites and for the cell surface stability of these proteins in neurons [55, 87]. Keller et al. [55] found that DHHC3, is a novel gamma2 subunit-interacting protein that results in the palmitoylation of the gamma2 subunit in a cytoplasmic loop domain-dependent manner. This mechanism is likely important for the regulation of GABAAR trafficking in the secretory pathway. Gephyrin is an anchoring protein found at inhibitory synapses, where it clusters glycine and subsets of GABAARs [38]. Recently, it was shown that gephyrin is palmitoylated by DHHC12, which facilitates the membrane association of gephyrin and regulates the size of postsynaptic gephyrin clusters [18]. The palmitoylation of gephyrin is an important mechanism underlying the increased strength of GABAergic synaptic transmission.

Most excitatory and inhibitory neurotransmitter receptors are reportedly concentrated at the PSD [11, 61]. Therefore, the PSD is the primary postsynaptic site that contributes to information processing. PSD protein-95 (PSD-95) is a scaffolding and hub protein that resides within the PSD at excitatory synapses and is the most abundant scaffold protein that regulates synaptic transmission and maturation by interacting with, stabilizing, and trafficking glutamate receptors, cytoskeletal elements, and cell adhesion molecules, respectively [14]. Previous studies have demonstrated that the synaptic localization of PSD-95 can be regulated through various PTMs, among which palmitoylation of PSD-95 is necessary for its participation in synaptic transmission and the formation of PSD-95 clusters at the postsynaptic membrane [15, 25]. An earlier study revealed that an increase in palmitoylated PSD-95 led to its accumulation at synaptic sites, implying that palmitoylation is crucial for its trafficking to synaptic sites to exert its functions [78]. The DHHC2, DHHC3, DHHC7, and DHHC15 subfamilies are PATs for PSD-95 in neurons [28]. Conversely, the depalmitoylation of PSD-95 mediated by a depalmitoylating enzyme (PPT) can be triggered by glutamate-induced synaptic activity, which consequently causes the dissociation of PSD-95 from synaptic sites [25]. Interestingly, several studies have shown that the palmitoylation–depalmitoylation cycle of PSD-95 contributes bidirectionally to AMPARs homeostasis [13, 25, 50]. PSD-95 palmitoylation has a critical role in the recruitment of synaptic AMPARs to specific synaptic sites [25, 78]. The increased palmitoylation of PSD-95 is associated with increased surface levels of AMPARs [13, 50]. Depalmitoylation of PSD-95 also causes endocytosis of AMPARs [25]. Therefore, whether the dynamic modification of PSD-95 could modulate the presentation and internalization of AMPARs by regulating their palmitoylation–depalmitoylation cycle is an interesting topic for future research.

### Roles of palmitoylation of other proteins in regulating synaptic proteins

#### GRIP1, AKAP79/150, and PICK1

In addition to the palmitoylation of synaptic proteins, palmitoylation of other proteins, which interact with synaptic proteins, can also affect synaptic transmission and function. Experimental evidence indicates that several proteins that directly or indirectly interact with AMPARs can also be important regulatory factors for trafficking and function of AMPARs [40]. The glutamate receptor-interacting protein (GRIP1)/AMPAR-binding protein (ABP), GRIP1, and ABP (also known as GRIP2), are multi-PDZ domain proteins that interact with the GluA2 and GluA3 subunits of AMPARs, which are involved in anchoring the intracellular or synaptic location of AMPARs [20]. Previous studies demonstrate that palmitoylated GRIP1 by DHHC5/8 is targeted to trafficking endosomes [111]. GRIP1 palmitoylation also plays specific roles in the regulation of endocytosis and trafficking of AMPARs under NMDARs activation conditions, implying a complex protein interaction that accelerates AMPARs recycling [41]. The A-kinase anchoring protein 79/150 (AKAP79/150) is a type of scaffold protein that targets various kinases to regulate AMPARs phosphorylation, thereby controlling AMPAR trafficking. Palmitoylation of AKAP79/150 has modulatory effects on its postsynaptic nanoscale organization, trafficking, and mobility in hippocampal neurons [10]. Importantly, AKAP79/150 palmitoylation reportedly regulates LTP-associated AMPAR trafficking pathways [54]. Protein interacting with C kinase 1 (PICK1) is a PDZ domain-containing protein that directly interacts with GluA2 and GluA3 of synaptic AMPARs [103]. PICK1 is a substrate of DHHC8, and DHHC8-dependent palmitoylation of PICK1 contributes to the internalization of postsynaptic GluA2-containing AMPARs, which are essential for the induction of long-term synaptic depression (LTD) in neurons [105, 112]. Until now, there very little evidence has been presented on how palmitoylated PICK1 and AKAP79/150 contribute to AMPAR trafficking and synaptic functions.

#### δ-Catenin and cyclin Y

Regarding the central role of PSD-95 in anchoring AMPARs and NMDARs to postsynaptic sites, the palmitoylation of proteins interacting with PSD-95 may also be important in maintaining synaptic function. δ-catenin is a cytosolic molecule that interacts with PSD-95 [51] and GRIP1b [99], and it has been shown that δ-catenin is palmitoylated by both DHHC5 and DHHC20 [4]. Brigidi et al. [4] reported that the palmitoylation of δ-catenin by DHHC5 mediates the surface insertion of AMPARs via direct interactions with the scaffold molecules PSD-95 and GRIP/ABP. Cyclin Y is a molecule enriched in the postsynaptic compartment. Palmitoylation of cyclin Y is required for its postsynaptic localization near PSD, regulates the synaptic surface expression of AMPARs and PSD-95, and influences AMPAR-mediated synaptic transmission [97].

#### AZ crucial presynaptic proteins

In the presynaptic compartment, the AZ contains crucial presynaptic proteins, in addition to the SNARE complex. Emerging evidence suggests that the AZ is a sophisticated structure that is composed of several evolutionarily conserved proteins, RIM, RIM-BP, Munc13, CAST/ELKS, bassoon, piccolo, and liprin-α [108]. These relatively large AZ proteins form a large macromolecular complex that docks and primes SVs, recruits voltage-gated Ca^2+^ channels to the docked and primed SVs, and tethers Ca^2+^ channels and SVs to synaptic cell-adhesion molecules [108]. Although the components of AZ are crucial presynaptic proteins during SVs exocytotic fusion, palmitoylation of AZ proteins has rarely been reported. In addition, current research is mainly performed to confirm the involvement of SNARE protein palmitoylation in SVs exocytotic fusion; however, it is not clear whether the specific functions of these proteins are altered after palmitoylation. Therefore, future research should focus on the importance of palmitoylation in regulating the more specific function(s) of presynaptic proteins.

## Association between palmitoylation of synaptic proteins and neurodegenerative diseases

Protein palmitoylation plays crucial roles in many physiological aspects of neurotransmission and synaptic functions in the mature nervous system; thus, it is not surprising that mutation/dysregulation of DHHC proteins and defects in the palmitoylation step of synaptic proteins commonly result in brain pathologies related to neurodegenerative diseases [16]. In this review, we aim to shed light on mutated and/or dysregulated DHHC proteins as well as the disrupted palmitoylation/depalmitoylation of synaptic proteins in neurodegenerative diseases such as AD, in particular, and Huntington’s disease (HD). However, there are only a limited number of studies investigating the effects of DHHC protein deficiency on the palmitoylation of synaptic proteins in the pathogenesis of neurodegenerative diseases.

### Alzheimer’s disease (AD)

AD is one of the most common neurodegenerative diseases characterized by progressive memory loss and cognitive decline. It is evident that several DHHC proteins are mutated in AD, which contribute to pathophysiology of AD [2, 63, 71]. Li et al. [62] reported a variant (c.999A > T, p.T209S) of *DHHC21* in a Han Chinese family with a familial Alzheimer’s disease (FAD) pedigree. The novel heterozygous missense mutation (p.T209S) was noted in all family individuals with AD with considerable beta-amyloid (Aβ) retention. Using a DHHC21^T209S/T209S^ knock-in mouse model, they found that the DHHC21 mutation contributes to AD pathology by inducing aberrant palmitoylation of FYN and APP, ultimately resulting in cognitive impairment [62]. A recent study reported that the levels of DHHC7 and DHHC21 are increased in the hippocampus of 3xTg-AD mice (a triple transgenic AD mouse model, expressing human gene mutants APPswe, PS1M146V, and tauP301L) and induce aberrant palmitoylation of key proteins that trigger Aβ aggregation in the brains of humans with AD [3, 30]. The DHHC12 is an APP-interacting DHHC protein and has been demonstrated to strongly inhibit APP metabolism, including Aβ generation, implying that DHHC12 may be involved in the early pathogenesis of AD [71]. However, it has not yet been investigated whether *DHHC12* is mutated or its expression level is altered in AD.

Over the last decade, several studies have revealed the roles of palmitoylation/depalmitoylation in neurodegenerative diseases, with a particular focus on AD [12]. Elevated levels of the Aβ peptide have been strongly implicated in the pathophysiology of AD. A recent study has found that Aβ modifies the conformation of the CTD of NMDARs, leading to synaptic weakening [21]. Importantly, PSD-95 has been previously found to be reduced in the brain tissues of AD mice [98]. Dore and coworkers have demonstrated that increasing the levels of synaptic PSD-95 by blocking its depalmitoylation or inducing overexpression of PSD-95 rescues Aβ-induced deleterious effects on synapses including the NMDAR CTD conformation and synaptic weakening [21]. Furthermore, it has also been shown that PSD-95 palmitoylation is essential for the protection of synapses from Aβ toxicity [21]. Collectively, selective pharmacological inhibition of PSD-95 de-palmitoylation may serve as a potential therapeutic avenue for AD.

### Huntington’s disease (HD)

HD is an autosomal dominant disease characterized by mutations in the huntingtin (*HTT*) gene. HTT is an important palmitoylated protein. It has been hypothesized that HD is caused by aberrant palmitoylation of HTT [94]. DHHC17 and DHHC13, also known as huntingtin-interacting protein 14 (HIP14) and huntingtin-interacting protein 14 like (HIP14 L), respectively, were found to induce palmitoylation of HTT at cysteine 214 (C214) [60]. Previous studies have documented that the loss of DHHC17 and DHHC13 leads to reduced palmitoylation of HTT, resulting in neuropathological deficits that are features of HD [94]. Beyond HTT, the synaptic proteins SNAP25 and AMPAR subunit GluA1 are substrates of DHHC17 [47]. Significant decreases in the palmitoylation of SNAP25 and GluA1 have been found in mice with disrupted DHHC17 enzymatic activity [48]; however, the association between reduced levels of SNAP25 and GluA1 palmitoylation and the pathogenesis of HD is not yet well understood. As a DHHC17 substrate, PSD95 synaptic targeting is palmitoylation dependent. In HD, PSD95 reduction at the postsynaptic site is associated with the loss of thalamostriatal excitatory synapses. Therefore, reduced PSD95 palmitoylation might also contribute to HD pathophysiology [49, 127]. In addition, the mutant HTT leads to both reduced DHHC17 interaction and activity, potentially resulting in reduced palmitoylation and DHHC17 substrate (e.g., GAD65, SNAP25, SYT1, and PSD95) mislocalization, ultimately aggravating HD pathogenesis [48, 90, 100, 124].

### Parkinson’s disease (PD)

Currently, there is a limited understanding of the genetic link between DHHC proteins and PD pathogenesis. However, palmitoylation of synaptic proteins influences the aggregation of pathological proteins in PD. SYT11 is one of 17 members of the synaptotagmin family, which is a group of transmembrane vesicle-associated proteins involved in regulating vesicle trafficking and endo-/exocytosis at various intracellular locations [120]. The functions of SYT11 in mediating vesicle trafficking and recycling are found to be shared by the neuronal protein α-synuclein, a key player in PD [27]. Ho et al*.* [45] found that SYT11 is palmitoylated in neurons, and that this modification targets SYT11 to digitonin-insoluble portions of membranes and protects it from endolysosomal degradation, thereby enhancing the binding of α-synuclein to intracellular membranes and promoting pathological α-synuclein aggregation in PD.

### Other neurodegenerative diseases

Evidence suggests that *DHHC8* is a schizophrenia susceptibility gene [86], and the identified *DHHC8* SNP rs175174 contributes to the risk of schizophrenia [74]. Several lines of evidence point to the role of DHHC8 in the pathogenesis of schizophrenia [26]. Defects in DHHC8 affect palmitoylation and cause a decrease in the number of dendritic spines, which may be a possible mechanism in the development of schizophrenia. However, the underlying mechanisms remain unclear. Loss-of-function mutations in *DHHC9* have been identified in patients with X-linked intellectual disability (XLID) [88]. The *DHHC9* mutation inactivates its PAT activity, affecting palmitoylation of target proteins, which is associated with language and memory impairment in XLID [58, 70]. In the future, the effects of these mutated DHHC proteins on the palmitoylation of targeted synaptic proteins should be investigated, which will be important for future understanding and treatment of these diseases.

## Future propects

Neurodegenerative disorders pose a global threat to human health. These diseases share many abnormalities in the fundamental processes that result in progressive neuronal dysfunction and death. An increasing number of studies have revealed the role of palmitoylation in various aspects of neuronal function. In particular, palmitoylation of synaptic proteins is crucial for neurotransmission and synaptic function, and dysregulated synaptic protein palmitoylation is linked to several neurodegenerative diseases, such as AD, HD, and PD. To date, the extent to which palmitoylation of these proteins contributes to pathophysiology is not completely understood and requires further investigation. Additionally, PATs and depalmitoylating enzymes (APTs and PPTs) are major regulators of palmitoylation and depalmitoylation. As demonstrated by several in vitro and in vivo models (e.g., DHHC-deficient mice), mutated and dysregulated DHHC proteins are associated with abnormal palmitoylation/depalmitoylation, potentially contributing to human neurological disorders in various cases [62, 71, 73]. However, the exact mechanisms underlying dysregulated palmitoylation/depalmitoylation in neurodegenerative diseases remain unclear. A better understanding of the link between aberrant palmitoylation of targeted synaptic proteins and the pathogenesis of neurodegenerative diseases may be helpful for developing therapeutic approaches in the future. Palmitoylation is a reversible PTM that mainly occurs on protein cysteine residues, and palmitoylated proteins are closely associated with membrane localization. We suggest that dysregulated palmitoylation/depalmitoylation may be related to diverse cellular homeostatic processes, such as redox and metabolic homeostasis.

An increasing number of studies have shown that palmitoylation of synaptic proteins is linked to synaptic function. Palmitoylation of presynaptic proteins, particularly the SNARE complex, regulates SV exocytotic fusion. Palmitoylation of postsynaptic receptors, including AMPARs and NMDARs, regulates their localization, transport, and clustering. In addition, palmitoylation of proteins at synaptic sites is quite complex, potentially regulating the function and homeostasis of other synaptic proteins. For instance, the palmitoylation–depalmitoylation cycle of PSD-95 contributes bidirectionally to AMPARs homeostasis. However, the functional protein pools associated with the palmitoylation–depalmitoylation cycle for protein trafficking, maturation, assembly, and dynamic changes should be fully elucidated in the future.

## Conclusions

This review compiles current knowledge regarding the role of palmitoylation in neurotransmission and how aberration in this process may lead to dysfunction and ultimately neurodegenerative conditions. Although many studies have identified mutations and/or dysregulation of DHHC proteins in several neurodegenerative diseases, research focused on discovering more potential disease-related DHHC proteins will throw further light of the detailed and specific mechanisms underlying the involvement of palmitoylation in the pathophysiology. Although the palmitoylation of certain synaptic key proteins has been extensively studied, the palmitoylation of other synapse-associated and synaptic function-regulating proteins is less commonly investigated. Therefore, the number of studies on DHHC family substrates is relatively disproportional, hampering our understanding of the associations between DHHC protein disruption and neurodegenerative disease pathogenesis. Future research should expand to the substrates of the DHHC family members, particularly DHHC proteins that are highly expressed in the brain cells mentioned in the present review. Uncovering DHHC proteins involved in the palmitoylation of important synaptic proteins is of critical importance.

### Supplementary Information

Additional file 1

## Data Availability

Not applicable.

## Abbreviations

PAT: Palmitoyl acyltransferases
SV: Synaptic vesicles
SNAREs: Soluble *N*-ethylmaleimide-sensitive-factor attachment receptor proteins
SYT: Synaptotagmins
PSD: Postsynaptic density
PTM: Posttranslational modification
