# Supplementary material for: Palmitoylation of synaptic proteins: roles in functional regulation and pathogenesis of neurodegenerative diseases
Source: Cell Mol Biol Lett. 2024 Aug 10;29:108. doi: 10.1186/s11658-024-00625-2 (PMC11316366; doi:10.1186/s11658-024-00625-2)
Supplement: Supplementary file 1 — Additional file 1 [file 11658_2024_625_MOESM1_ESM.docx]

**Table S1 Primer sequences for qPCR.**

| **Gene** | **Forward (5`- 3`)** | **Reverse (5`- 3`)** |
| --- | --- | --- |
| DHHC1 | CCATCTTCAACCGCAGCCAGCAT | CCAGGAGCAGGACACCCAATAAAGC |
| DHHC2 | ACCAGACCGCTGTCATCATTGTTCC | ACTTGGCTTGAGTATCAGGCAGACC |
| DHHC3 | TCATCCTGCTCATCCTGCTGTGCTT | TCTGCCTTCCCTTGGTCTGGTGTG |
| DHHC4 | TGCCACCATAGCCACCGTGACT | CACAAGTAGCCAGCCAGGAGCAT |
| DHHC5 | CCAGGTTACAGCAGCAGTAGCACAT | GAGGATCGTGAGCCACTGGACAATG |
| DHHC6 | CCTGGATCAACAACTGCTGTGGTCA | GCAAACAAGGTGGCGGCAAACG |
| DHHC7 | AGCACCATCCTCTCCTGACTGACAA | GAGCACCGCCAAGCAGTTGAAGA |
| DHHC8 | GAGGACGAGGACAAGGAGGATGACT | CTGAGTGACAGCAGGAACAGGAAGA |
| DHHC9 | ACAGCCACGGCAACATTGTGAAGAA | TGCTGGTGTCCTCTGCCATCTCATT |
| DHHC11 | TCGTGATGCTTGGGCACCTCCT | CGGCGAAAGAGTAGACACTGGGAAC |
| DHHC12 | CCACCTTCCTGCTGCTCTCCTTCT | GCTGCTGCCTTCTTCCTCTTCCTC |
| DHHC13 | AGCATCCATCTGGCGGTCCTGTT | CTGCTGCAACTGCCCAGTGAAGAG |
| DHHC14 | CACTCCTGATGAAGCCGCTGATCTG | CACAGTTGCCTACCCAGGGACAGT |
| DHHC15 | CCGCTGCCATCACTGTTCTGTCTG | GCAGGCCACAAAGAGGAGAAAGAGG |
| DHHC16 | CCAGACTCCACCACCTACCTTCTCC | CCTTCCAGTTGTCCAAGCAGCCATA |
| DHHC17 | ACTGTGAGACCACGTACACCAAGGA | AAGTGCTTGTATCTCCTGGCGTTCA |
| DHHC18 | CTCATCCTCAGCACCACCATCCTCT | TCGGTATGTGGAGCTGCCTGTGTTA |
| DHHC19 | TGCTGTGACACTTGTGAAGGAACCA | TGAGCGAGACGAGACTGAAGAAGGT |
| DHHC20 | ACCTTTGTGGTCGTCTGGTCCTACT | TGCTGTCTTTCTTGGCTGAACTCCT |
| DHHC21 | GGTTGGTGCTGCATGGGCTTGA | GCTCTGCATGTGGGATCTTGGGATT |
| DHHC22 | GCTCTTCTCGCCTGCTGTGCTT | ATGGTCGTGCCTCAGCGTGACT |
| DHHC23 | CCGCACACTGAAGGACGATGTTAGG | TGATGGTTTGATTCTCCGACGCAGC |
| DHHC24 | GTGTGCATCCTACGTCGTGACCATC | GAGTAGCATGAGCCAGGGCAACAG |
| GAPDH | ACGGCAAATTCAACGGCACAGTCA | GGTCTCGCTCCTGGAAGATGGTGAT |
